# DNA methylation patterns of β-globin cluster in β-thalassemia patients

**DOI:** 10.1186/s13148-020-00987-2

**Published:** 2020-12-03

**Authors:** Xiuqin Bao, Yangjin Zuo, Diyu Chen, Cunyou Zhao

**Affiliations:** 1 Department of Medical Genetics, School of Basic Medical Sciences, Guangdong Technology and Engineering Research Center for Molecular Diagnostics of Human Genetic Diseases, and Guangdong Engineering and Technology Research Center for Genetic Testing, Southern Medical University, Guangzhou, 510515 China; 2 Laboratory of Genetics and Metabolism, Maternal and Child Health Hospital of Guangxi Zhuang Autonomous Region, Nanning, China; 3 Key Laboratory of Mental Health of the Ministry of Education, Guangdong-Hong Kong-Macao Greater Bay Area Center for Brain Science and Brain-Inspired Intelligence, and Guangdong Province Key Laboratory of Psychiatric Disorders, Southern Medical University, Guangzhou, Guangdong China

**Keywords:** β^0^ thalassemia patients, β-globin cluster, Fetal hemoglobin, Methylation patterns, Bone marrow, Cord blood, Developmental stage

## Abstract

**Background:**

Reactivation of fetal hemoglobin (HbF, α_2_γ_2_) holds a therapeutic target for β-thalassemia and sickle cell disease. Although many HbF regulators have been identified, the methylation patterns in β-globin cluster driving the fetal-to-adult hemoglobin switch remains to be determined.

**Results:**

Here, we evaluated DNA methylation patterns of the β-globin cluster from peripheral bloods of 105 β^0^/β^0^ thalassemia patients and 44 normal controls. We also recruited 15 bone marrows and 4 cord blood samples for further evaluation. We identified that the CpG sites in the locus control region (LCR) DNase I hypersensitive site 4 and 3 (HS4-3) regions, and γ- and β-globin promoters displayed hypomethylation in β^0^/β^0^-thalassemia patients, especially for the patients with high HbF level, as compared with normal controls. Furthermore, hypomethylations in most of CpG sites of the HS4-3 core regions were also observed in bone marrows (BM) of β^0^/β^0^-patients compared with normal controls; and methylation level of γ-globin promoter -50 and + 17 CpG sites showed lower methylation level in patients with high HbF level compared with those with low HbF level and a negative correlation with HbF level among β^0^-thalassemia patients. Finally, γ-globin promoter + 17 and + 50 CpG sites also displayed significant hypomethylation in cord blood (CB) tissues compared with BM tissues from normal controls.

**Conclusions:**

Our findings revealed methylation patterns in β-globin cluster associated with β^0^ thalassemia disease and γ-globin expression, contributed to understand the epigenetic modification in β^0^ thalassemia patients and provided candidate targets for the therapies of β-hemoglobinopathies.

## Background

The human β-globin cluster, spanning a 70-kb region, is composed of five genes (5′-*HBE* -*HBG2* -*HBG1* -*HBD* -*HBB*-3′; 5′-ε-γ^G^-γ^A^-δ-β-3′) and a distal regulatory element known as LCR [1], which plays an important role in regulating the expression of all the genes in the cluster. β-thalassemia is characterized by a quantitative defect in the synthesis of β-globin chains underlaid by a marked genotypic heterogeneity of β-globin gene mutations. Reactivating of HbF was reported to be a therapeutic target for β-thalassemia and SCD. Several genetic modulators [2–7] and *cis*-regulatory elements [6, 8–12] involved in the regulation of human fetal hemoglobin (HbF), and concomitant α-thalassemia have been identified as ameliorators of β-thalassemia [13]. DNA methyltransferase (DNMT) inhibitor 5-azacytidine (5-aza) has been applied to hemoglobinopathy patients (mainly in SCD patients) through improvement of HbF expression in the 1980s and 1990s [14, 15], which supported that DNA methylation plays an important role in human globin switching. In 2007, Rodwell Mabaera et al. used three human fetal liver and three BM samples, respectively, to reveal the changes of DNA methylation in β- and γ-globin promoters during fetal-adult globin switching, and proved the negative correlation between promoters DNA methylation level and expression of globin genes in human development stage [16]. Mei Hsu et al. have analyzed the methylation status of regions in the murine β-like globin locus in uncultured primitive and definitive erythroblasts and other cultured primary and transformed cell types [17]. They found a 20-kb domain, extended from the region just past the LCR to before β-major and encompassed the embryonic genes εy, βh1, and βh0, is hypomethylated only in primitive erythroid cells. Even retrotransposons in this region are hypomethylated in primitive erythroid cells.

Although epigenetic-based underlying mechanism driving the fetal-to-adult hemoglobin switch have been studied and reported in mouse and human, the systematic scanning of methylation patterns in β-globin cluster remains to be determined, especially in β-thalassemia patients. Here, we uncovered the methylation patterns of β-globin cluster, and explored its roles in regulating HbF gene expression in PB, BM, or CB samples from human β-thalassemia patients or normal controls.

## Results

**Fig. 1:**
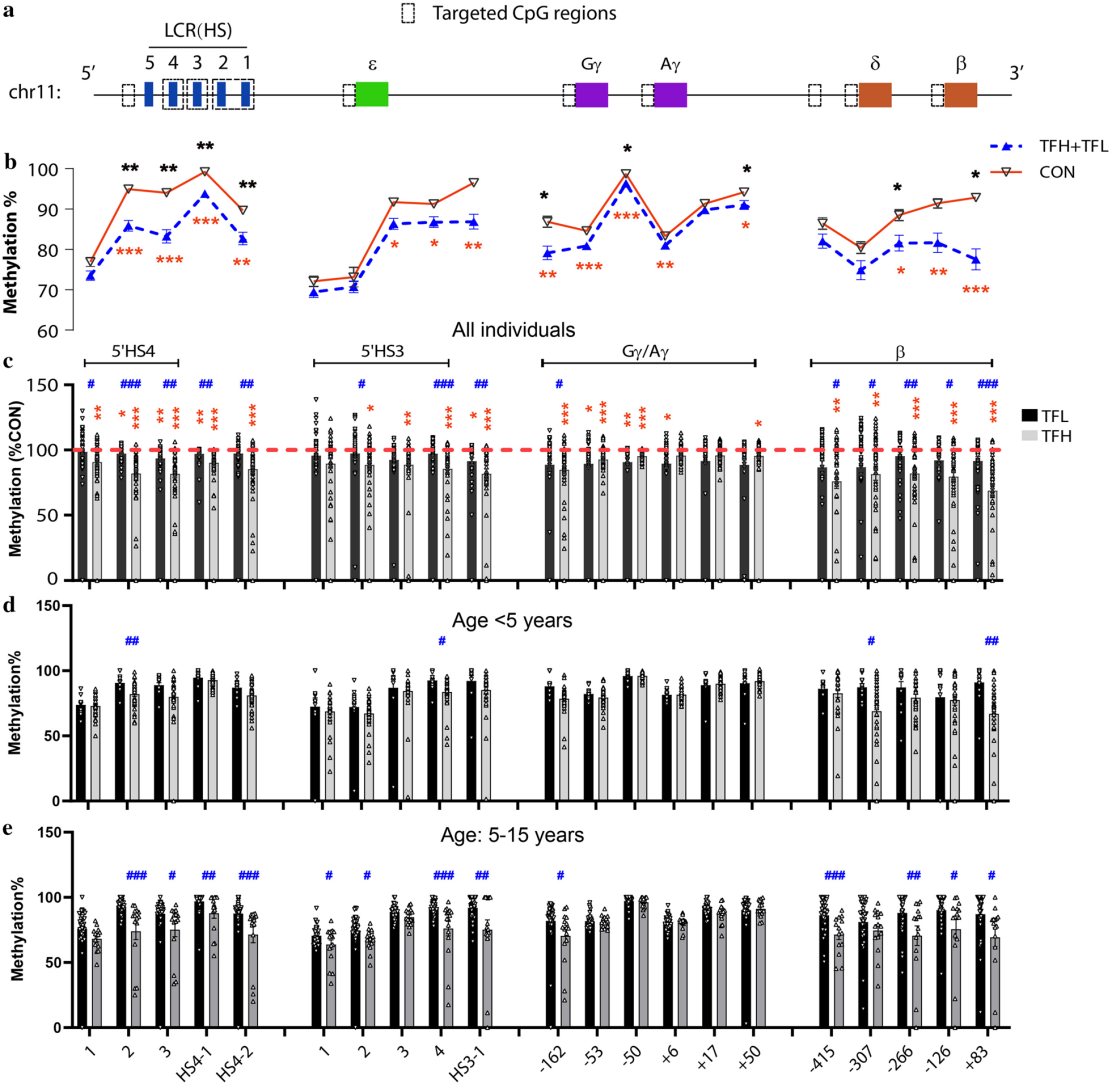
Methylation patterns of β-globin cluster in PB tissues. **a** Schematic of β-globin cluster**.** The dashed box showed the target regions in DNA methylation examination. **b** Methylation differences in β^0^-thalasssmia patients from normal controls (CON) were shown with *p < 0.05, **p < 0.01, ***p < 0.001 in ANCOVA analysis without (under indicated sites) or with (above indicated sites) age covariate. **c** Methylation level relative to CON (the horizontal dashed line) in TFL (black) or TFH (gray) patients are represented by scatter plot for each CpG site with standard error indicated by bars. *p < 0.05, **p < 0.01, ***p < 0.001 indicated the significant difference in the TFH and/or TFL patients from CON. **d**, **e** Methylation pattern between the TFH and TFL patients with age < 5 years (**d**) and age within 5–15 years (**e**). ^#^*p* < 0.05, ^##^*p* < 0.01, ^###^*p* < 0.001 indicated significant difference from *t *test between the TFH and THL group in panels of **c** to **e**. Five CpG sites (2631 for site 1, 2753 for 2, 2786 for 3, 2868 for HS4-1 and 2928 for HS4-2 relative to the first nucleotide of LCR (chr11:5275850, hg38)) around HS4, five CpG sites (5808 for 1, 5860 for 2, 5943 for 3, 6093 for 4 and 6164 for HS3-1 relative to the first nucleotide of LCR) around HS3, six CpG sites (− 162, − 53, − 50, + 6, + 17 and + 50 relative to the transcriptional start site of γ-globin gene) in the γ-promoter and five CpG sites (− 415, − 307, − 266, − 126 and + 83 relative to the transcriptional start site of β-globin gene) in the β-promoter are shown under each column in panel **e**

To evaluate the methylation status of the β-globin clusters in β-thalassemia patients, we first screened the methylation patterns of total 97 CpG sites on the β-globin cluster, including ε-, γ-, δ-, and β-globin promoters, the core sequences of HS4 and HS3 and the 5′ flanking sequences of the LCR region, the CpGs-rich region of the HS2-HS1 intergenic region, the CpG islands (CpGI) in the endogenous retrovirus 9 (ERV-9) located at upstream of HS5, and the predicted binding region of the transcription factor BCL11A on the gene cluster located about 3.5 kb upstream of δ-globin gene [9] (Fig. 1a), in PB of 105 β^0^/β^0^ thalassemia patients and 44 normal controls using bisulfite sequencing method as previously described [12]. We observed that most of CpG sites around the LCR HS4-HS3 regions, and γ- and β-globin promoter displayed significant hypomethylation in β^0^/β^0^-thalassemia patients compared with normal controls (Fig. 1b, shown with stars under indicated sites), especially for the TFH patients (Fig. 1c). We didn’t find any significant differences in methylation level in the other examined regions among the three indicated groups (data not shown). Since DNA methylation can be associated with aging progression and significant differences in age between β-thalassemia patients and normal controls, we then included age as a covariate in analysis of covariance (ANCOVA) of methylation difference between β-thalassemia patients and normal controls and observed that hypomethylation in β-thalassemia patients remained significance in most of CpG sites around the LCR HS4 region, and γ- and β-globin promoters (Fig. 1b, shown with stars above indicated sites). We further divided the β^0^/β^0^ thalassemia patients into two subgroups: subjects with age < 5 years old (Fig. 1d) and subjects with 5–15 years old (Fig. 1e), and observed that the TFH patients still displayed significant lower methylation levels than the TFL patients, especially for the subjects with 5–15 years old (Fig. 1e).

**Fig. 2:**
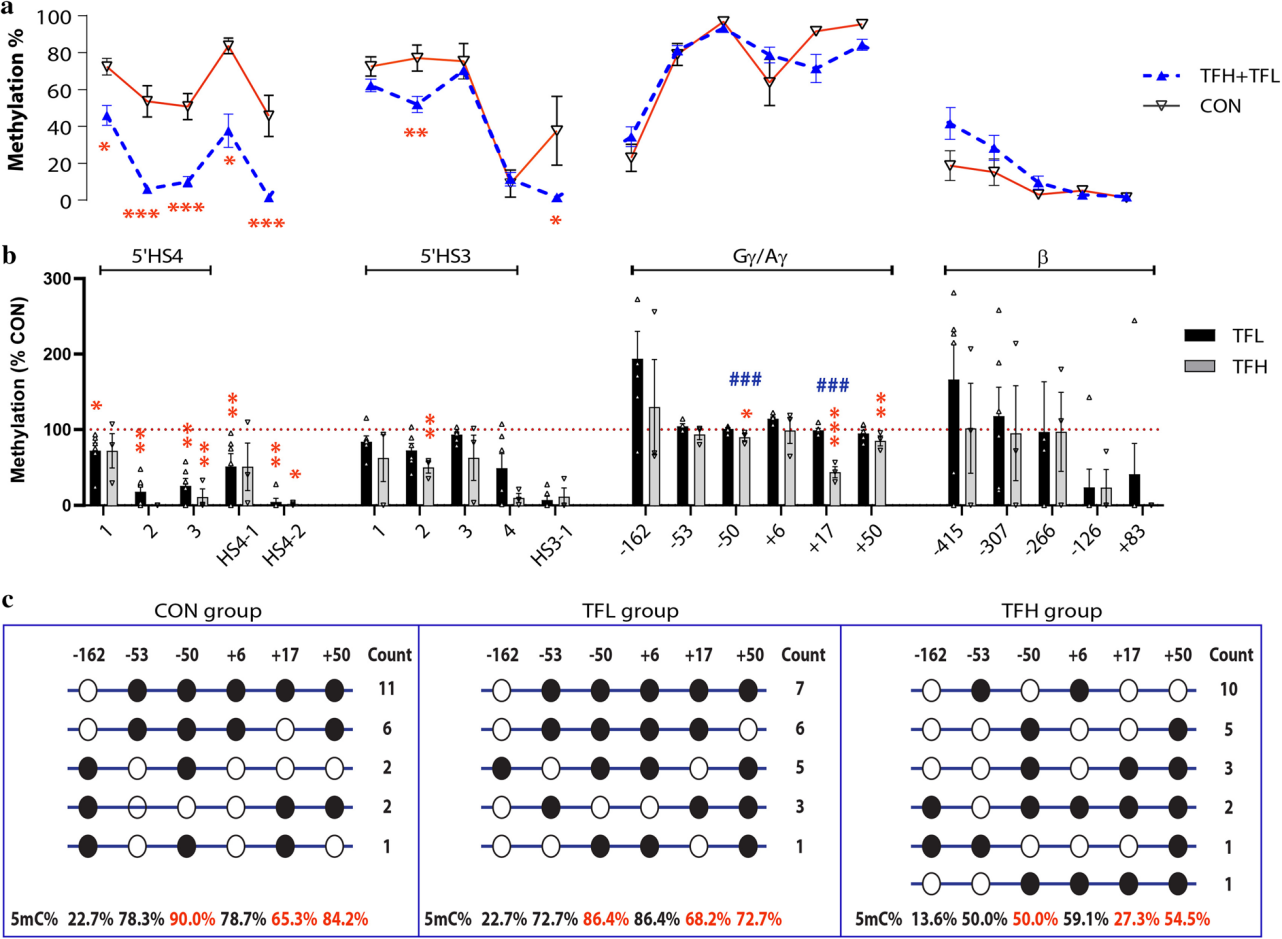
Methylation patterns of β-globin cluster in BM tissues. **a** Methylation differences in β-thalassemia patients from CON were shown with *p < 0.05, **p < 0.01 and ***p < 0.001 from *t-*test. **b** Methylation level relative to CON (the horizontal dashed line) in TFL (black) or TFH (gray) patients are represented by scatter plot for each CpG site with standard error indicated by bars. *p < 0.05, **p < 0.01, ***p < 0.001 indicated the significant difference in TFH and/or TFL from CON. ^###^*p* < 0.001 indicated significant difference between TFH and THL group. **c** Methylation levels at CpG sites in *HBG* promoter verified by bisulfite-treated clone sequencing. ● methylated CpGs; ○ unmethylated CpGs. Count, the numbers of the same clone

Given that DNA in PB mainly derived from the lymphocyte cells, which will have some limitations for DNA methylation research with high tissue heterogeneity, we then examined DNA methylation in BM GYPA (Glycophorin A) positive cells and observed that significant hypomethylation at most of CpG sites in core regions of HS4-HS3 and the 5′ flanking sequences, especially in the HS4 region, in β^0^/β^0^-patients compared with the normal controls (Fig. 2a). Moreover, we observed that the TFH patients showed significant lower methylation level at the − 50 and + 17 CpG sites of γ-globin promoter than both the TFL patients and normal controls (Fig. 2b). We did not observe any significant differences in β-globin promoter between β^0^/β^0^-patients and normal controls. Furthermore, we employed bisulfite clone method to validate methylation pattern of γ-globin promoter in β^0^/β^0^-thalassemia patients and normal controls, and observed that hypomethylation at − 50, + 17, and + 50 CpG sites of γ-globin promoter in the TFH patients compared with the TFL patients and normal controls (Fig. 2c).

**Fig. 3:**
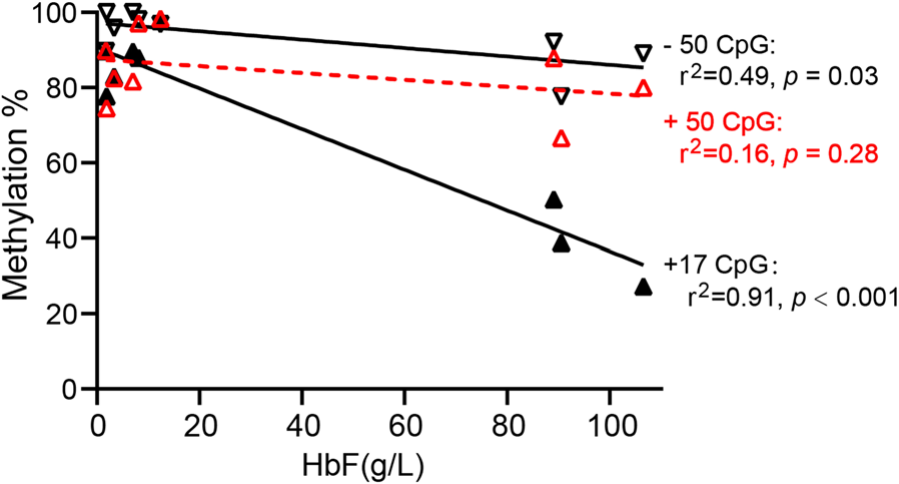
Relationship between γ-globin promoter DNA methylation level and HbF level. Relationships between γ-globin promoter − 50, + 17, and + 50 CpG sites DNA methylation level and HbF level in BM tissues of nine β^0^/β^0^-patients were represented by lines shown with correlation coefficient *r*^2^ and *p* value

**Fig. 4:**
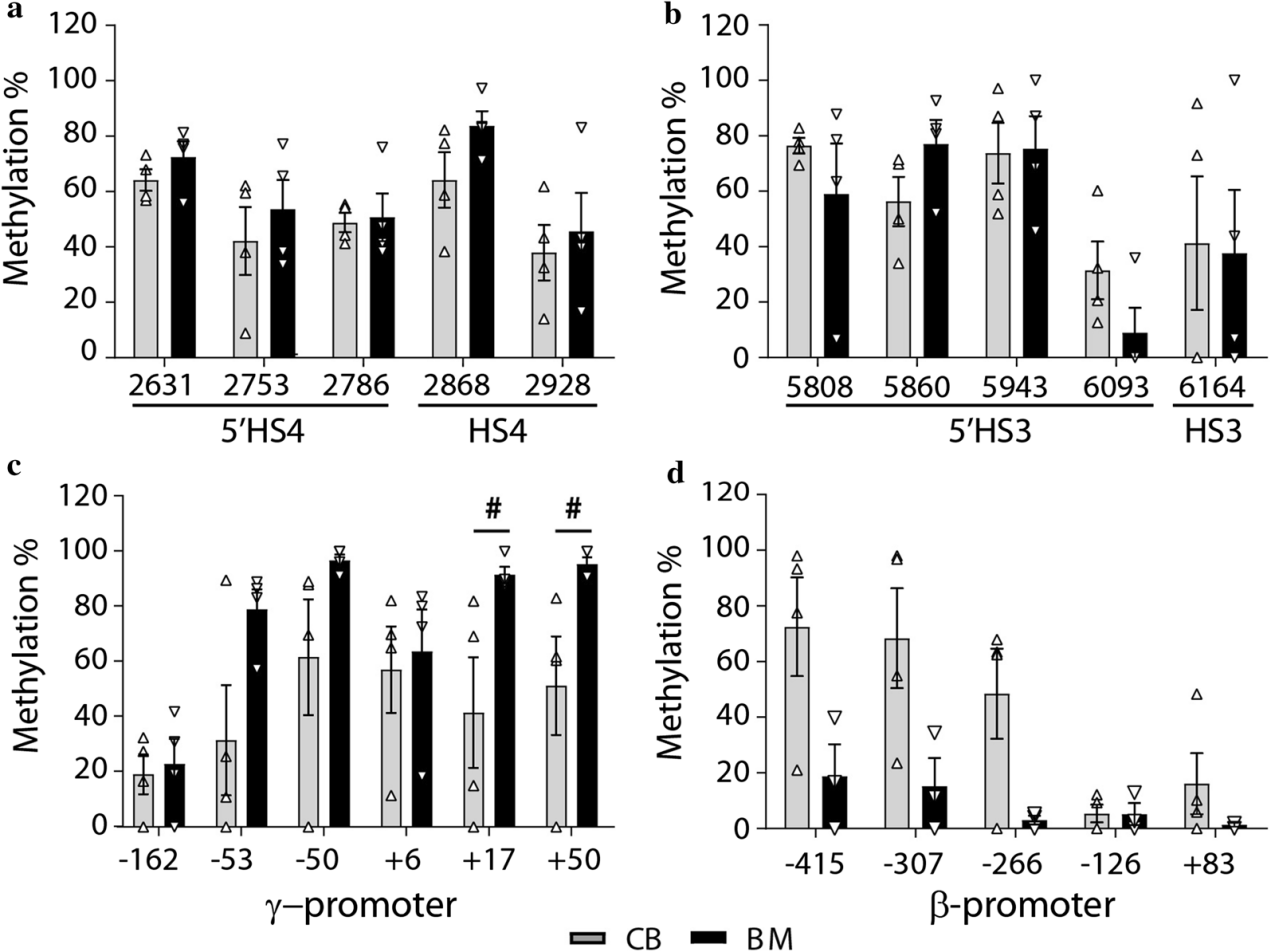
DNA methylation patterns in CM and BM tissues. Methylation levels of the indicated CpG sites in the LCR 5′HS4 and the cord region of HS4 (**a**), 5′HS3 and the cord region of HS3 (**b**), γ-globin promoter (**c**), and β-globin promoter (**d**) between CB tissue (grey) and BM (black) tissues of normal controls are shown in columns with standard error indicated by bars. ^#^*p* < 0.05 from two-tailed *t*-test

To further verify the relationship of the methylation status of γ-globin promoter with the HbF level, we performed Pearson correlation analysis of γ-promoter methylation level with HbF level in BM tissues from nine β^0^/β^0^-patients and observed that γ-globin promoter—50 (*r*^2^ = 0.49, *p* = 0.03) and + 17 (*r*^2^ = 0.91, *p* < 0.001) CpG sites displayed significantly negative correlations with the HbF level (Fig. 3). More importantly, we observed that γ-globin promoter + 17 and + 50 CpG sites displayed significant hypomethylation in CB tissues compared with BM tissues of normal controls, which is consistent with a higher HbF expression level in CB (HbF: 76.2 ± 7.9%; Fig. 4) than in BM (HbF: 0.3 ± 0.1%). Altogether, these results indicated that the methylation level of LCR HS4-3 regions was associated with thalassemia, and the methylation level of γ-globin promoter was associated with HbF expression.

## Discussion

In this study, we found that the hypomethylation in LCR regions, especially the HS4-3 core regions, was correlated with thalassemia; the methylation level of + 17 CpG site in γ-globin promoter was negatively correlated with HbF expression level, providing an important role of methylation in regulating globin expression in different developmental stage.

It is important to uncover the methylation patterns that account for phenotypic manifestation and HbF expression in β-thalassemia. A previously study has uncovered the methylation patterns in CD34^+^ primary cells from fetal liver and BM of healthy donors [18]. They found that several differentially methylated CpGs are located near genes with unanticipated roles in red blood cell differentiation and proliferation, and a correlation between the γ-globin promoter DNA methylation and HbF levels in red blood cell progenitors, which was consistent with our results that observed in BM. Some researchers also discovered dynamic DNA methylation of γ-globin promoter in mouse uncultured primitive, definitive erythroblasts, and other cultured primary and transformed cell types, supporting a mechanistic role for DNA methylation in developmental regulation of globin genes [17]. This dynamic DNA methylation pattern was further observed in our study that γ-globin promoter methylation was higher in BM than in CB tissue, elucidating the negative relationship between γ-globin promoter methylation and gene expression with developmental stage.

We further found that the methylation patterns in LCR regions from PB tissue were mostly hypomethylated in β^0^/β^0^-thalassemia patients, which was consistent with the case-controls difference patterns observed in BM tissue. However, we found the methylation patterns in γ- globin promoter showed difference between TFH and TFL group of β-thalassemia. The methylation level of most CpG sites in γ-globin promoter were hypomethylated, especially in + 17 and + 50 sites, in the BM tissues of TFH patients, compared with TFL patients, which was consistent with our previous report [12]. The γ-globin promoter methylation, not LCR region DNA methylation, might be the main effect to γ-globin expression, and the methylation alteration in the γ-globin promoter may be a driving factor leading to remote regulation between globin promoter and LCR regions [19]. The hypomethylated patterns in globin promoters might make it easier to access for some transcription factors mediating the looping with globin promoter. In fact, our previous study showed that a SNP rs368698783 was associated with the methylation level on + 17 CpG site in γ-globin promoter [12], by recruiting DNMT3A to the promoter and further regulating the γ-globin expression, supporting the important role of γ-globin promoter methylation in regulation of γ-globin expression.

Since only 15 BM subjects employed in this study to obtain methylation alterations in LCR regions associated with β^0^-thalassemia and in γ-globin promoter associated with HbF expression level, it will require larger samples from BM tissues to further verify these results. In addition, given the limitation of ethic approval, we have no evaluated the patterns in fetal liver, which would give a more comprehensive view of the methylation patterns in different developmental stage. Finally, effect of methylation alterations at γ-globin promoter + 17 CpG site on the HbF level should be examined by using site-specific methylation through dCas9-MQ1-sgRNA system in further study.

## Conclusion

Our findings revealed methylation patterns in β-globin cluster associated with β^0^ thalassemia disease or γ-globin expression, contributed to understand the epigenetic modification in β^0^ thalassemia patients and provided candidate targets for the therapies of β-hemoglobinopathies.

## Methods

### Human subjects

We recruited 105 subjects with β^0^/β^0^-thalassemia from southern China. These 105 unrelated Chinese β-thalassemia patients with a large range of HbF expression levels were stratified into low- and high-HbF groups for this study: 52 cases of high HbF (TFH, HbF: 58.9 ± 19.8%) and 53 cases of low HbF (TFL, HbF: 2.0 ± 0.7%). A subset of 44 healthy adult controls (CON, HbF: 0.3 ± 0.2%) was selected from a large random Guangxi population screened as the previously mentioned [20] (Additional file 1: Tables S1 and S2). DNA extracted from peripheral blood lymphocyte (PB) of above 105 β^0^/β^0^ thalassemia patients and 44 healthy donors were used for DNA methylation analysis. Moreover, DNA extracted from GYPA (Glycophorin A) positive cells separated from bone marrows (BM) of nine β^0^/β^0^ thalassemia patients including six TFL β^0^/β^0^-patients (HbF: 7.46 ± 5.28 g/L) and three TFH β^0^/β^0^-patients (HbF: 95.39 ± 9.69 g/L) and six healthy adult controls (HbF: 0.37 ± 0.05 g/L), eight of which were also used in our previous study [12], and from CB (HbF: 76.2 ± 7.9%) of four healthy adult controls were used for examination of the methylation patterns in different tissues.

Hematological parameters were measured using an automated hematology analyzer (Sysmex F-820; Sysmex Co. Ltd., Kobe, JP). Adult and fetal hemoglobin analysis was carried out using high-performance liquid chromatography (Variant II, Bio-Rad Laboratories, Hercules, CA). DNA was extracted DNA by Phenol–chloroform method. We used XmnI PCR-restriction fragment length polymorphism (RFLP) and high-resolution melting (HRM) assays to genotype *KLF1* mutations and three primary HbF-associated single nucleotide polymorphisms (rs7482144, XmnI, in *HBG2*, rs766432 in *BCL11A*, and rs9399137 in *HBS1L-MYB*) as described [4, 12] to unified genetic variants between TFH and TFL group. The study protocol was reviewed and approved by the local medical ethics committee at Nanfang Hospital, China, and conducted in accordance with the Declaration of Helsinki.

### DNA methylation assay

We used bisulfite conversion method to examine DNA methylation as previously described [12]. Bisulfite conversion of DNA was performed by denaturing 1 mg genomic DNA with 0.3 M NaOH at 42℃ for 20 min, followed by 95℃ for 3 min and 0℃ for 1 min, and incubating at pH 5.0 with sodium metabisulfite (2.0 M) and hydroquinone (0.5 mM) at 55℃ for 16 h in the dark overlaid with mineral oil. Modified DNA was purified with Promega Wizard DNA Clean Up System (Madison, WI, USA). The eluted DNA was incubated with NaOH (0.3 M) at 37℃ for 15 min and neutralized by 3 M NH_4_-acetate to pH 7.0. The neutralized DNA was precipitated by 75% ethanol and recovered in 20 ml of TE buffer. We used nested-PCR to amplify the target DNA regions and performed Sanger sequencing. Because unmethylated cytosine (C) was replaced by thymine (T) after PCR reaction, any presence of C at CpG dinucleotides on the sequence represented a methylated C allele in the bisulfite-modified DNA. The percentage methylation of CpG sites was determined based on the C and T allele peaks by means of BioEdit Sequence Alignment Editor v7.0.9.0 (Carlsbad, CA, USA). We further performed bisulfite-treated clone sequencing assay to verify DNA methylation patterns examined by Sanger sequencing. In brief, we cloned BS-PCR products of bisulfite-DNA into the pMD19T cloning vector (D104, TAKARA, Japan), and sequenced 22 clones to calculate the percentage of C as methylation% for each subject. The primers used in this study are listed in Additional file 1: Table S3.

### Statistical analysis

A two-tailed student *t*-test from SPSS v20 software was used for comparisons between the two indicated groups in this study. ANCOVA analysis with age as a covariate was also used in case–control analysis of methylation difference. *P* values of < 0.05 were considered as statistically significant.

## Supplementary information

**Additional file 1:** Phenotypic data of human subjects (Tables S1 and S2) and sequences of primers (Table S3) employed in this study.

## Data Availability

All the data in this study were available in the figures and tables in the manuscript.

## Abbreviations

HbF: Fetal hemoglobin
PB: Peripheral bloods
BM: Bone marrow
SCD: Sickle cell disease
LCR: Locus control region
HS4-3: Hypersensitive site 4 and 3
DNMT: DNA methyltransferase
5-aza: 5-Azacytidine
CpGI: CpG islands
ERV-9: Endogenous retrovirus 9
Glycophorin A: GYPA
CB: Cord blood
RFLP: Fragment length polymorphism
HRM: High-resolution melting
