# Supplementary material for: DNA methylation patterns of β-globin cluster in β-thalassemia patients
Source: Clin Epigenetics. 2020 Dec 3;12:187. doi: 10.1186/s13148-020-00987-2 (PMC7712619; doi:10.1186/s13148-020-00987-2)
Supplement: Supplementary file 1 — Additional file 1: Phenotypic data of human subjects (Tables S1 and S2) and sequences of primers (Table S3) employed in this study. [file 13148_2020_987_MOESM1_ESM.docx]

**Supplemental materials for**

**DNA methylation patterns of β-globin cluster in β^0^-thalassemia patients**

Xiuqin Bao^1^, Yangjin Zuo^1,2^, Diyu Chen^1^, Cunyou Zhao^1,*^

**Table S1. The phenotypic data of 149 PB samples.**

|  | TFH (52) | TFL (53) | CON (44) | P value |
| --- | --- | --- | --- | --- |
| Age (years) | 4.7±5.1 | 7.6±3.8 | 22.3±2.0 | 0.000 |
| Gender (F: M) | 35:17 | 31:22 | 23:21 | 0.286 |
| Age of onset (months) | 3.8±2.8 | 4.0±2.4 | / | 0.739^#^ |
| Transfusions (/y) | 5.7±6.3 | 14.5±5.9 | / | 0.000^#^ |
| Hb (g/L) | 62.4±17.5 | 76.2±19.3 | 138.1±15.9 | 0.000 |
| HbF (%) | 58.9±19.8 | 2.0±0.7 | 0.3± 0.2 | 0.000^#^ |
| HbA2 (%) | 2.9±1.1 | 2.7±0.4 | 2.8±0.2 | 0.298 |
| MCV (fl) | 75.5±5.5 | 85.4±5.2 | 89±5.6 | 0.000 |
| MCH (pg) | 23.4±2.5 | 27.9±2.0 | 30.5±3.0 | 0.000 |

HbA2: hemoglobin A2; MCV: mean corpuscular volume; MCH: mean corpuscular hemoglobin

Characteristics of the patients were compared among the TFH group, TFL and CON group with the Pearson chi-square test for categorical variables.

Data were shown as mean ± SD.

^#^P value showed the difference between TFH and TFL group.

**Table S2. The phenotypic data of 15 BM samples.**

|  | TFH (3) | TFL (6) | CON (6) | P value |
| --- | --- | --- | --- | --- |
| Age (years) | 17.0±8.18 | 7.8±1.2 | 17.5±1.6 | 0.001 |
| Gender (F: M) | 1:2 | 1:5 | 0:6 | 0.364 |
| Age of onset (months) | 8.0±1.0 | 0.5±0.2 | / | 0.000^#^ |
| Transfusions (/y) | 0.0±0.0 | 15.1±3.6 | / | 0.000^#^ |
| Hb (g/L) | 99.3±11.8 | 88.0±9.4 | 153.2±16.9 | 0.000 |
| HbF (%) | 95.4±9.7 | 5.7±4.2 | 0.3±0.1 | 0.000 |
| HbA2 (%) | 3.8±2.1 | 2.9±0.2 | 3.1±0.3 | 0.361 |
| MCV (fl) | 78.9±1.8 | 74.2±9.1 | 89.7±3.3 | 0.004 |
| MCH (pg) | 24.5±2.2 | 23.0±4.7 | 31.5±1.0 | 0.002 |

HbA2: hemoglobin A2; MCV: mean corpuscular volume; MCH: mean corpuscular hemoglobin.

Characteristics of the patients were compared among the TFH group, TFL and CON group with the Pearson chi-square test for categorical variables. ^#^two-paired t test was used for comparison between TFH and TFL group.

Data were shown as mean ± SD.

**Table S3. The primers used in this study.**

| **Target** | **Primers** | **Forward primer (5’-3’)** | **Reverse primer (3’-5’)** |
| --- | --- | --- | --- |
| 5’HS4 and HS4 | EL1 1st | TTTTTGTATTAGTGGTTTTTTGAAGAG | AATTATCATCTTAAAACCTCAACTTTC |
|  | EL1 2nd | TTTGGGAATAGTTAAATAGTAATT | CAATATTACAAAATTAATCTAACAAT |
| 3’HS4 | EL2 1st | TAGTAGTTATGAGATGGTTTGTTTTG1F | TACTCTTAATCTCTACTCTATACTACCC |
|  | EL2 2nd | TTAGGTTGATTTATTTTAAGGTTT | AAAAATACTAAATTCTTTCCTAAAAA |
|  | EL3 1st | TGGAGTTAGATTATTTGAGTGATTAGTG | AAAACACTAATTTTCACCTACAACAAA |
|  | EL3 2nd | GGAGGGTTTTTTAATTAGTTTGT | AACAAAAAATACTAACAAACATTTCT |
| 5’HS3 | EL4 1st | AAGGGTTTGGAAAATTTGTGAGTTT | ATTTCTCAAAAAAACCATTCTCCCCA |
|  | EL4 2nd | TTTATAGGGGATTTTTATTTTGAGTA | AATCATCTCAACAAAATTCAAAAAAATA |
| HS3 and 3’HS3 | EL5 1st | TTGTATTGATGAGGAGGATTAGATGGA | AAAAATCATAAACTCCACCCAACACCA |
|  | EL5 2nd | TAATGAAGTAAAGGAATTATTTTGTGG | ACCTATAACCCATCTAAACCCTAAT |
|  | EL6 1st | TTATTGTTAGAGATATATTGTTAGTTTT | CAATCTTAACTCACTACAACCTCC |
|  | EL6 2nd | GAGTTTGATGTTAATTTAGTAATGGG | CCTCCTAAATTCAAATAATTCTCCTA |
| 5’HS2 | EL7 1st | GTTTGGTATGGTGGTGTGTA | CATCACTCTAAACTAAAAACATCTAAAC |
|  | EL7 2nd | GGTGTGTA TTTGTAATTTTAGT | ACACCCTAAACCTCAACATAAC |
| HS2 and 3’HS2 | EL8 1st | GAGTTATGATGAGTTATGTTGA | CTTCACAATTACCCACACAAATAAA |
|  | EL8 2nd | GTTTAGAGTGA TGATTTTTATTTGGG | ATCTTCCTAATACACCAAAACTAACC |
| HS1 | EL9 1st | TTTGTGTTTATGGATTATTGG | ACACTAACAACTTAAATACATAATTA |
|  | EL9 2nd | TTAGTTAAATTTTTGGAATGTA | TTTTTTATTCTATTACAATTATATACTA |
| *HBG* | 1st | TTAAAAATTTTGGATTTATGTTTA | CAAATTACCAAAACTATCAAAAAACC |
|  | 2nd | TTAAATTATAGGTTTTATTGGAGTT | AATCAAAAAATACCACAAATCC |
| *HBB* | 1st | TAAGAAAAATAATAATAAATGAATGTA | TCTCCACATACCCAATTTCTATTAATC |
|  | 2nd | ATTAGAAGGTTTTAATTTAAATAAGGA | ACCTTAATACCAACCTACCCAAAAC |
| *BCL11A* | rs766432 | CGCTTCTCAGACCCAAATGCTC | GGCTTTCTAGACTGGTGGACG |
| *HBS1L-MYB* | rs9399137 | CGGTTCCCTCAGAAGACACTTAC | TTCACTGCCAGAAGCACTTTGG |
